# Optimization of Phage-Antibiotic Combinations against Staphylococcus aureus Biofilms

**DOI:** 10.1128/spectrum.04918-22

**Published:** 2023-05-18

**Authors:** Razieh Kebriaei, Susan M. Lehman, Rahi M. Shah, Kyle C. Stamper, Ashlan J. Kunz Coyne, Dana Holger, Amer El Ghali, Michael J. Rybak

**Affiliations:** a Anti-Infective Research Laboratory, College of Pharmacy and Health Sciences, Wayne State University, Detroit, Michigan, USA; b Center for Biologics Evaluation and Research, U.S. Food and Drug Administration, Silver Spring, Maryland, USA; c School of Medicine, Wayne State University, Detroit, Michigan, USA; The University of North Carolina at Chapel Hill

**Keywords:** MRSA, bacteriophages, biofilms

## Abstract

Phage therapy has gained attention due to the spread of antibiotic-resistant bacteria and narrow pipeline of novel antibiotics. Phage cocktails are hypothesized to slow the overall development of resistance by challenging the bacteria with more than one phage. Here, we have used a combination of plate-, planktonic-, and biofilm-based screening assays to try to identify phage-antibiotic combinations that will eradicate preformed biofilms of Staphylococcus aureus strains that are otherwise difficult to kill. We have focused on methicillin-resistant S aureus (MRSA) strains and their daptomycin-nonsusceptible vancomycin-intermediate (DNS-VISA) derivatives to understand whether the phage-antibiotic interactions are altered by the changes associated with evolution from MRSA to DNS-VISA (which is known to occur in patients receiving antibiotic therapy). We evaluated the host range and cross-resistance patterns of five obligately lytic S. aureus myophages to select a three-phage cocktail. We screened these phages for their activity against 24-h bead biofilms and found that biofilms of two strains, D712 (DNS-VISA) and 8014 (MRSA), were the most resistant to killing by single phages. Specifically, even initial phage concentrations of 10^7^ PFU per well could not prevent visible regrowth of bacteria from the treated biofilms. However, when we treated biofilms of the same two strains with phage-antibiotic combinations, we prevented bacterial regrowth when using up to 4 orders of magnitude less phage and antibiotic concentrations that were lower than our measured minimum biofilm inhibitory concentration. We did not see a consistent association between phage activity and the evolution of DNS-VISA genotypes in this small number of bacterial strains.

**IMPORTANCE** The extracellular polymeric matrix of biofilms presents an impediment to antibiotic diffusion, facilitating the emergence of multidrug-resistant populations. While most phage cocktails are designed for the planktonic state of bacteria, it is important to take the biofilm mode of growth (the predominant mode of bacterial growth in nature) into consideration, as it is unclear how interactions between any specific phage and its bacterial hosts will depend on the physical properties of the growth environment. In addition, the extent of bacterial sensitivity to any given phage may vary from the planktonic to the biofilm state. Therefore, phage-containing treatments targeting biofilm infections such as catheters and prosthetic joint material may not be merely based on host range characteristics. Our results open avenues to new questions regarding phage-antibiotic treatment efficiency in the eradication of topologically structured biofilm settings and the extent of eradication efficacy relative to the single agents in biofilm populations.

## INTRODUCTION

Antibiotic-resistant infections take a staggering toll in the United States and across the world. Almost 2 million people in the United States develop hospital-acquired infections, which lead to 99,000 deaths annually, mainly associated with antibacterial-resistant pathogens (1–3). Bacteriophage (phage) therapy is utilizing bacterial viruses (the most abundant microorganisms on earth) as antibacterial agents against external and internal bacterial infections. Phage cocktails are frequently proposed for phage therapy (4). This strategy involves the application of more than one phage type and offers potential benefits such as a broader spectrum of activity and lower frequency of resistance (5, 6). One of the potential limits of phage therapy, specifically monophage therapy, is the development of resistance. Importantly, phage receptors, such as lipopolysaccharides, outer membrane proteins, cell wall teichoic acids, etc., may also be components of virulence factors, which may lead to trade-off costs against antibiotics leading to resensitization to antibiotics (7).

There are several reports describing the superior activity of phage-antibiotic combinations against planktonic bacteria (8–13). We have previously reported on the synergistic effect of phage Sb-1 in combination with antibiotics daptomycin (DAP), vancomycin (VAN), and ceftaroline (CPT) both in biofilm and planktonic bacteria (8). We noticed that the synergistic results achieved in planktonic bacteria were not necessarily translatable to the biofilm state. Therefore, here, we have focused on synergistic phage-antibiotic combinations against the biofilm state.

The purpose of this study was to (i) identify phage combinations with a broader host range and potentially lower frequency of resistance than single phages, (ii) test these combinations against biofilms of methicillin-resistant Staphylococcus aureus (MRSA), and (iii) evaluate the impact of phage-antibiotic combinations against biofilms of MRSA. The phages were selected from our existing libraries of sequenced, obligately lytic double-stranded DNA (dsDNA) S. aureus phages. The phage library included myophages belonging to two genera within the *Herelleviridae* family and *Twortvirinae* subfamily, as well as podophages belonging to a single genus within the *Rountreeviridae* family and *Rakietenvirinae* subfamily (per the taxonomy reflected in ICTV Master Species List number 37). A shortlist of 5 phages was chosen based on their collective host range and genetic diversity. None of the tested podophages were included in this shortlist because they had a very narrow host range and resistance arises very readily, presumably because of their specificity for β-glycosylated wall teichoic acid (WTA) over other forms of WTA (7). These phages were tested for their ability to infect spontaneously occurring phage-resistant bacterial mutants, and a cocktail of three phages was ultimately chosen based on a balance of these characteristics. The three selected phages were individually tested for their ability to inhibit biofilm. These biofilm experiments used strain pairs consisting of an MRSA or heteroresistant vancomycin-intermediate S. aureus (hVISA) parent strain and a derivative that is genetically identical except for the mutations that make it into a DNS-VISA strain. We then evaluated combinations of the phage cocktail with antibiotics against biofilms of two strains that were least sensitive to phages alone.

## RESULTS

### Phage cocktail selection.

**FIG 1:**
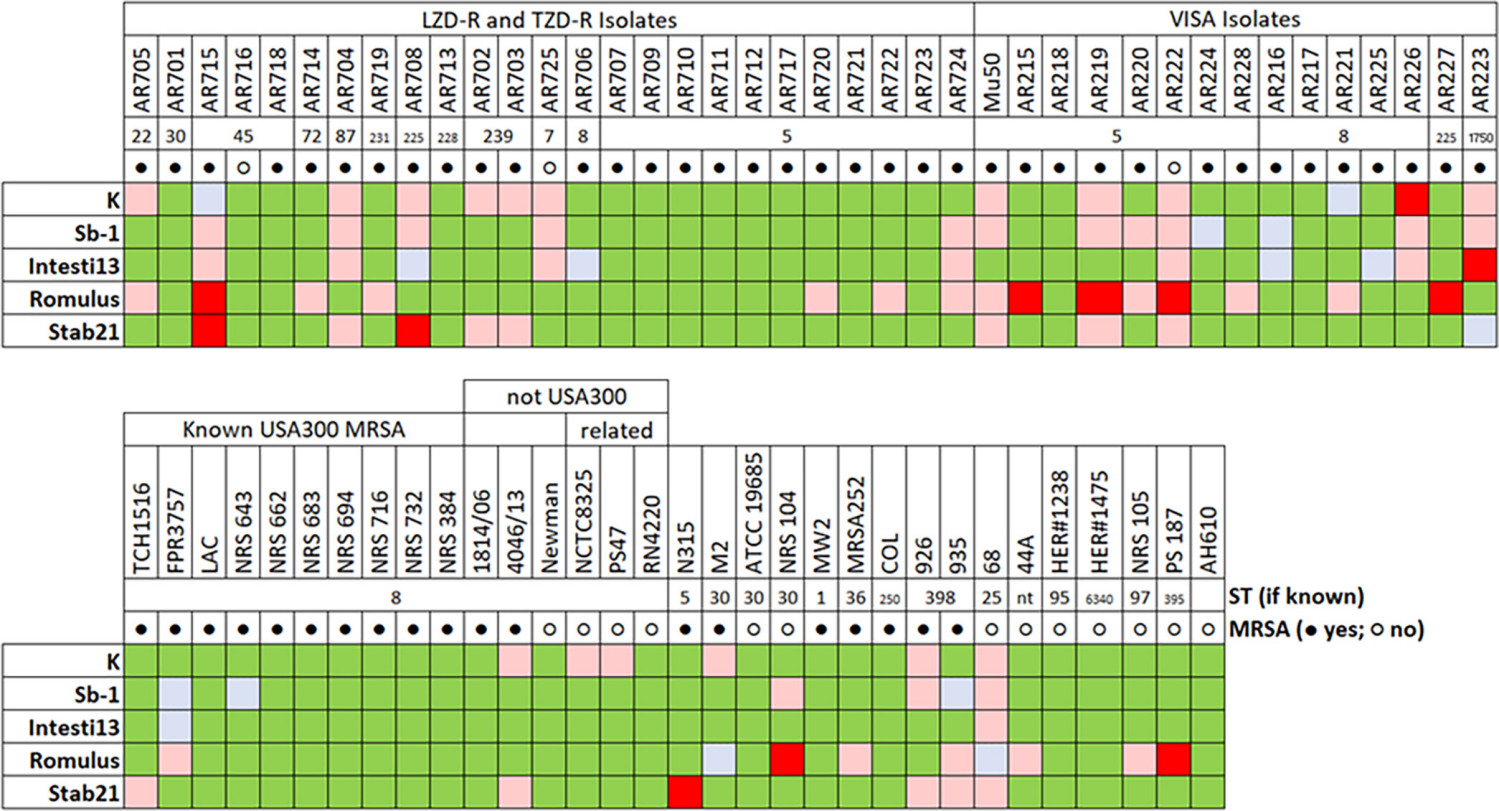
Host ranges of 5 phages on 72 S. aureus strains, as determined by dilution spotting on HIB agar. All phages were standardized to 1 × 10^8^ PFU/mL on their respective growth hosts. LZD-R, linezolid-resistant; TZD-R, tedizolid-resistant. Green cells indicate obvious productive infection (i.e., plaquing) by the main population of the tested phage. Blue-gray cells indicate very faint plaques that might not be discernible in full-plate titration. Only green and blue-gray cells were considered positive for phage infection. Pink cells indicate that the phage suspension had an effect on the bacteria at high concentration(s), but no plaques were observed as the phage was diluted, suggesting lysis without other phenomena. Red cells indicate no effect of phage at all.

Previous host range screening using 24 S. aureus phages and >70 S. aureus strains narrowed down our phage selection to the 5 phages that, taken together, yielded the broadest host range (plaquing on 71 of 72 strains) with the fewest phages (Fig. 1). These five phages were individually tested against some of the daptomycin-nonsusceptible vancomycin-intermediate (DNS-VISA) S. aureus or hVISA S. aureus strains being considered for subsequent phage-antibiotic synergy testing. Two main questions were explored using both plate and broth-based assessments, (i) which phages had activity against the MRSA and DNS-VISA strain pairs to be used for subsequent phage-antibiotic synergy testing, and (ii) if bacterial resistance to any of the phages could be countered by other phages.

**FIG 2:**
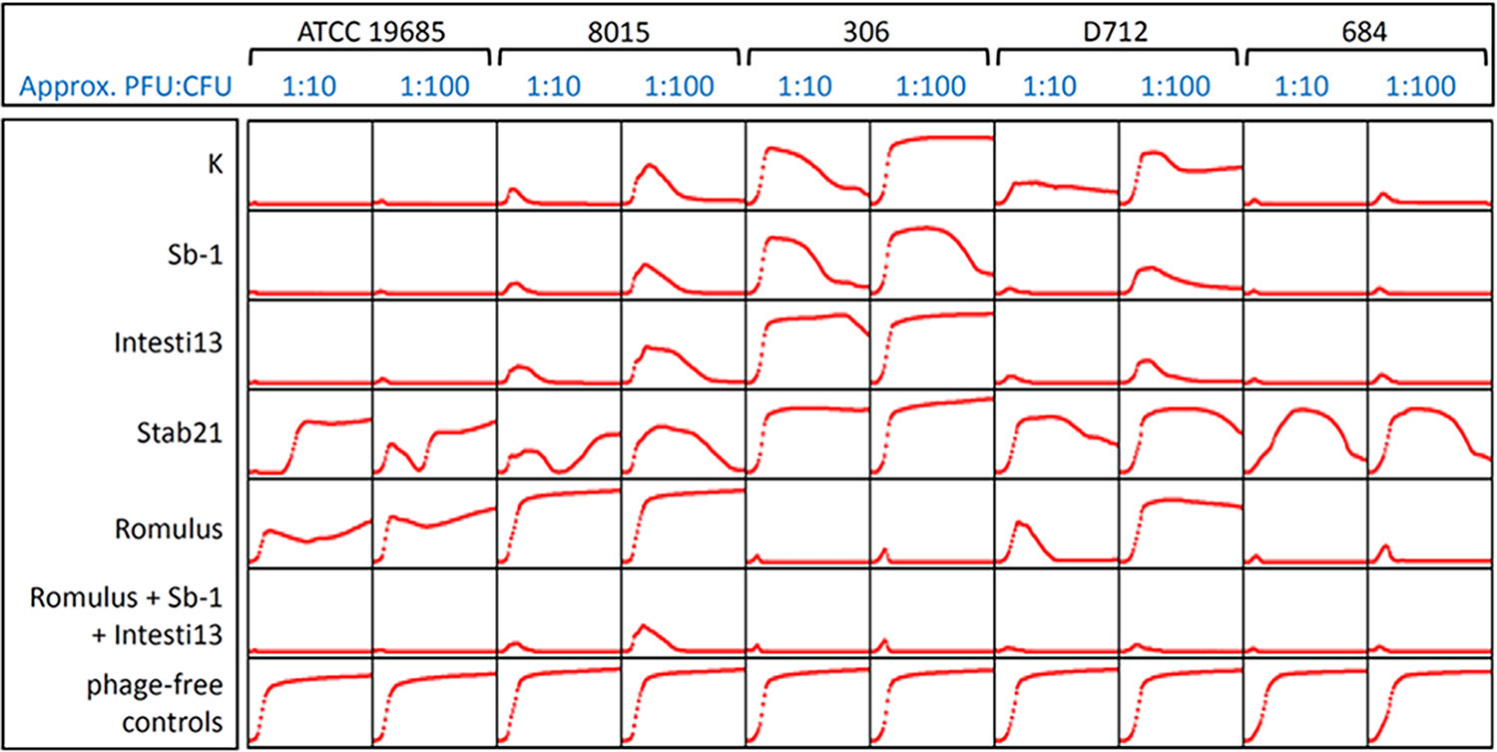
Phage activity on MSSA control (ATCC 19685) and DNS strains, as assessed by bacterial population suppression in broth. Each plot is a 48-h growth curve of OD_600_ versus time. PFU/CFU ratios are initial values at the time of plate inoculation.

In plate-based sensitivity tests (Table 1), phages K, Sb-1, Intesti13, and Stab21 plaqued equally well on the three tested strain pairs, whereas Romulus only formed plaques on one of the three strain pairs. Phage activity differed slightly when we investigated the ability of these phages to suppress planktonic bacterial growth in broth over 48 h. As indicated in the column headings of Fig. 2, phages were initially present in the broth at either 1/10th or 1/100th of the concentration of bacteria. In some cases, both concentrations of phage were able to suppress bacterial growth; in other cases, the lower concentration of phage was less effective (e.g., Romulus versus D712, or K, Sb-1, and Intesti13 versus 8015). The most notable differences between plaque and broth assays involved phage Stab21. For example, phages K, Sb-1, Intesti13, and Stab21 appeared to have equivalent activity in plaque assays (Table 1), but Stab21 was clearly less able to suppress bacterial growth in broth (Fig. 2). However, within this small sample of strains, there was no association between phage sensitivity in planktonic cells and the evolution from MRSA to DNS-VISA genotypes.

**TABLE 1:** Phage sensitivity of MSSA control and MRSA strains, as assessed by plaque formation^*a*^

Lineage of strain pair	Strain ID	Phenotype	Phage titer on each strain [log_10_(1 + PFU/mL)]				
			K	Sb-1	Intesti13	Stab21	Romulus
ST30 (control)	ATCC 19685	MSSA	**7.89**	**7.82**	**7.97**	**7.52**	**5.80**
USA100 ST5	D712	DNS-VISA	**7.83**	**7.73**	**8.00**	**7.26**	0.00
	D592	hVISA	**7.83**	**7.63**	**7.91**	**7.56**	0.00
USA100 ST5	8015	DNS-VISA	**7.73**	**7.76**	**7.86**	**7.50**	0.00
	8014	MRSA	**7.74**	**7.72**	**7.77**	**7.37**	0.00
USA300 ST8	306	DNS-VISA	**7.72**	**7.44**	**7.54**	**7.49**	**7.75**
	305	MRSA	**7.91**	**7.83**	**7.80**	**7.62**	**7.89**
USA100 ST5	684	DNS	**7.76**	**8.02**	**7.86**	**7.45**	**7.74**
	675	MRSA	**7.88**	**8.13**	**7.91**	**7.50**	**7.29**

a Values are the mean of three to five replicates. All standard deviations were ≤0.50. The titer of each phage was standardized to 1 × 10^8^ PFU/mL on its propagation host; therefore, values close to 8.0 indicate that the indicate the phage plaques on the listed S. aureus strain as well as its propagation host. Bold cells indicate obvious productive infection (i.e., plaquing) by the main population of the tested phage. Light gray shading indicates that the phage suspension had an effect on the bacteria at high concentration(s), but no plaques were observed as the phage was diluted, suggesting lysis from without other phenomena. Dark gray shading indicates no effect of phage at all.

Cross-resistance testing was conducted to understand which phage combinations might help prevent phage-resistant bacteria from arising. In order to isolate bacteriophage-insensitive mutants (BIMs) that spontaneously arise in the absence of phages, fresh cultures of the DNS-VISA strains were grown in heart infusion broth (HIB) and then exposed to an excess of each phage in an agar overlay. Colonies that appeared after 48 h of incubation at 37°C were counted as apparent BIMs, and a selection of these were subcultured for follow-up phage sensitivity testing. The frequency of BIMs (Table 2) was generally consistent with the plate-based host range data (Table 1), with discrete, countable BIMs being isolated from all phage-host pairings that yielded plaques in previous testing and confluent bacterial growth being observed when Romulus was mixed with the strains on which it did not plaque. The frequency of resistance experiment was only conducted once, to estimate the order of magnitude at which spontaneous resistance to these phages occurs *in vitro*. The values in Table 2 are comparable to previously reported frequencies for related phages (14). Some of the BIMs isolated in these experiments were sensitive to the original phage when rechallenged using the spot titer assay, suggesting that their growth on the BIM plate may have been the result of a transient tolerance phenotype (e.g., from gene expression changes, such as in reference 15) or simple evasion of phage by chance. BIMs that proved truly resistant to the original phage when rechallenged had various patterns of cross-resistance to the other 4 phages in our test set (see Tables S1 through S3 in the supplemental material). In general, cross-resistance to Romulus and any of the other phages was rare; cross-resistance among K, Sb-1, and Intesti13 was more common but did not always occur. BIM challenge experiments were also conducted in broth, and the cross-resistance results were generally consistent with plaque assay results (data not shown).

**TABLE 2:** Frequency of BIMs appearing after 48 h exposure to each phage^*a*^

Strain or phage	Frequency of BIMs appearing after 48-h exposure to:				
	K	Sb-1	Intesti13	Stab21	Romulus
ATCC 19685	2.2 × 10^−8^	<2 × 10^−8^	2.2 × 10^−8^	3.8 × 10^−6^	1.7 × 10^−6^
D712	1.5 × 10^−7^	1.2 × 10^−7^	ND	6.3 × 10^−6^	NA
8015	8.3 × 10^−8^	1.3 × 10^−7^	4.2 × 10^−8^	2.1 × 10^−7^	NA
306	4.2 × 10^−8^	2.3 × 10^−7^	5.2 × 10^−7^	4.6 × 10^−7^	<2 × 10^−8^

a In most pairings, a 10-fold excess of phages (or more) was used. Romulus plates were used a 2- to 5-fold excess. If no BIMs were observed, the frequency is shown as being less than the limit of detection for that strain based on the total number of cells that were plated. NA, the strain was not sensitive to the phage; ND, not determined.

To choose the final phage cocktail for this study, the following parameters were considered: (i) phages having broad but different host ranges (Fig. 1), (ii) evidence that spontaneous resistance to one phage does not always cause cross-resistance to the other phage(s) (Tables S1 through S3), and (iii) minimal genome similarity at least between 2 out of the 3 phages selected for this cocktail (Fig. S1). In this context, several observations emerged: (i) phage K has been reported to have poor activity against some bacterial strains at 37°C (16–18), although it had reasonable activity in these experiments; (ii) Sb-1 and Intesti13 had similar behavior in the BIMs tests but have different host ranges; (iii) Romulus had a more limited host range than the other phages; however, it was often able to kill bacteria that had become resistant to other phages; and (iv) Stab21 did not add much to cocktail host range or to BIM killing if Romulus and one of Sb-1 or Intesti13 were present. A 3-phage cocktail of Intesti13, Sb-1, and Romulus was therefore selected for our phage-antibiotic experiments. This cocktail was able to completely suppress bacterial growth by all strain pairs in Table 1. It was also able to prevent the planktonic growth of at least one BIM that could not be suppressed by any single phage (Fig. S3). We did not explicitly consider phage receptors in our choice of cocktail components because, unlike with most bacterial species, all known lytic S. aureus phages use WTA as their cell surface receptor, with the myophages typically being much less sensitive to biochemical modifications of WTA than the podophages (7, 19). We have therefore relied on the empirical data from BIM cross-resistance experiments to indicate which phages have complementary mechanisms of evading bacterial defenses.

### Antibiofilm activity of individual antibiotics and phages.

**FIG 3:**
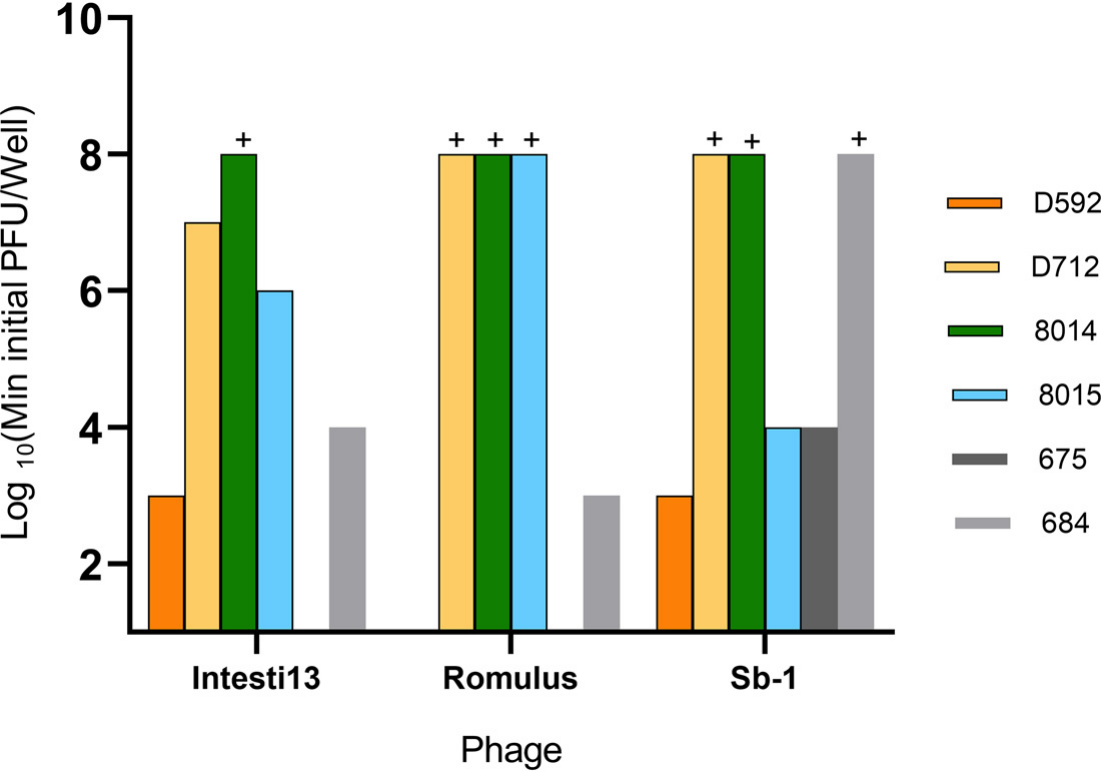
Minimum phage concentration required to prevent detectable bacterial regrowth from bead biofilms. All experiments are done in duplicate. The plus sign indicates that even the highest tested concentration of phage (10^7^ PFU/well) failed to prevent bacterial regrowth.

The strain pairs used during phage cocktail selection were chosen because they exhibited biofilm formation of ≥25% of our in-house reference strain in a crystal violet assay (Fig. S2). Fig. 3 shows the antibiofilm effects of single phages. To test this property, we grew 24-h bead biofilms, transferred the beads into fresh medium in 96-well plates, and exposed them to individual phages from our chosen cocktail in concentrations ranging from 10 PFU/well to 10^7^ PFU/well for 24 h. Wells that did not become turbid after 24 h of incubation were deemed a treatment success because bacterial growth was substantially inhibited. An inherent limitation of this experimental approach is that it did not quantify biofilm CFU and therefore does not support claims of biofilm eradication. It remains possible that bacteria survived or grew while remaining below the limit of detection offered by turbidity measurements. However, our prior experience has suggested that well turbidity is a reasonable proxy for more quantitative outcomes, making this a useful screening method for studies such as this one, in which testing multiple multiplicities of infection (MOIs), phages, and strains required more than 200 test combinations, all of which were run in duplicate.

Some of the strains (such as D592 and 675) demonstrated high sensitivity to single-phage treatments, and complete eradication of bacterial biofilms was observed with single phages at low concentrations. Within isogenic strain pairs, the DNS-VISA mutant was substantially less phage sensitive in one case (D712 versus D592), slightly less phage sensitive in one case (684 versus 675), and moderately more phage-sensitive in one case (8015 versus 8014). Minimal sensitivity to individual phages was our inclusion criteria for further investigating cotreatment with antibiotics and the phage cocktail. Therefore, additional investigation focused on D712 (DNS-VISA) and 8014 (MRSA), and minimum biofilm inhibitory concentrations (MBIC) of daptomycin, vancomycin, and ceftaroline were measured for these two strains (Table S4).

### Antibiofilm activity of phage-antibiotic combinations.

**FIG 4:**
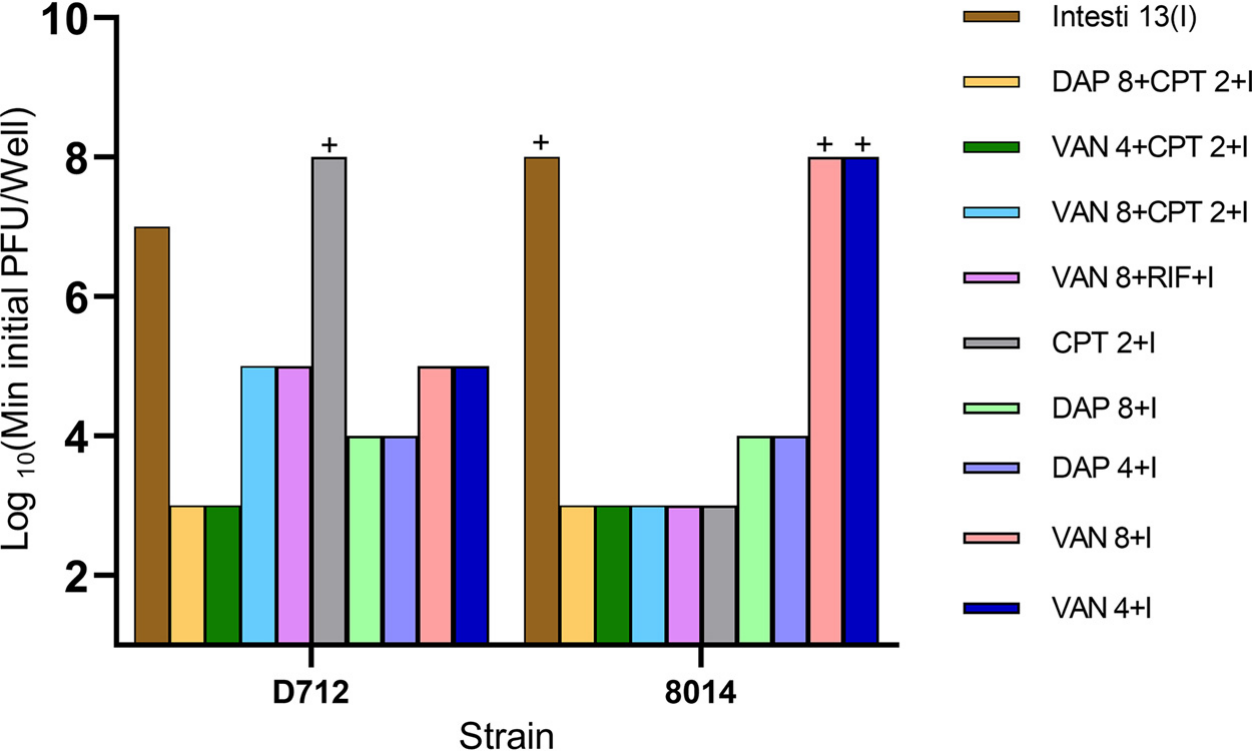
Minimum phage concentration required to prevent detectable bacterial regrowth from bead biofilms. All experiments were done in duplicate. The plus sign indicates that even the highest tested concentration of phage (10^7^ PFU/well) was unable to prevent bacterial regrowth. VAN, vancomycin; DAP, daptomycin; CPT, ceftaroline; RIF, rifampin. The combination of three phages, Intesti13, Sb-1, and Romulus, showed the same minimal phage concentration values. VAN4 and DAP4 refer to concentrations equal to half the MBIC. VAN8 and DAP8 refer to concentrations equal to minimum biofilm inhibitory concentration (MBIC) values, CPT2 refers to concentrations equal to MBIC, and I refers to Intesti13, which was present in all displayed treatment groups.

Antibiotics were added to phage treatments, using phage and antibiotic concentrations below or equal to those previously found to prevent bacterial regrowth. Results are shown in Fig. 4. Data are shown for Intesti13 alone and in combination with various antibiotics. Identical results were obtained with the 3-phage cocktail. Results combining antibiotics with Romulus or Sb-1 were generally poorer and are not shown. Regarding the DNS-VISA S. aureus D712, both Intesti13 and the 3-phage cocktail were effective at concentrations as low as 10^3^ PFU/well adjunctive to antibiotics. While single-phage treatment was only effective at 10^7^ PFU/well of Intesti13, the combination of vancomycin with ceftaroline or rifampin and either Intesti13 or the cocktail was effective at phage concentrations as low as 10^3^ PFU/well, implying phage-antibiotic synergy. Similarly, biofilms of the MRSA strain 8014 demonstrated no sensitivity to any of the single phages at concentrations as high as 10^7^ PFU/well but showed clearance of biofilms at concentrations as low as 10^3^ PFU/well with adjunctive phage-antibiotic treatments. Most combinations of Intesti13 (or phage cocktail) with antibiotics were more effective than the phage alone, except for phage with ceftaroline against D712 and phage with vancomycin against 8014.

## DISCUSSION

Phage-antibiotic combinations are a potential alternative against biofilm-forming pathogens. Existing in a compact biofilm structure can potentially increase the overall target size for phage-mediated predation, while phage-antibiotic combinations can potentially reduce resistance (16, 20, 21). Biofilms are potentially capable of using phenotypically phage-resistant cells to surround and protect sensitive cells (22). In addition, previous studies have shown that biofilm matrix-degrading enzymes, such as various hydrolytic enzymes, are encoded on phage genomes and can improve phage diffusion inside the matrix barrier (6, 9, 23). Our primary hypothesis was that the addition of antibiotics to the phages and phage cocktails would introduce an independent secondary killing mechanism to eradicate surviving phage-tolerant and -resistant populations. Previous microscopy data have shown that subinhibitory concentrations of Sb-1 can degrade the extracellular polysaccharide component of ATCC 43300, possibly due to the presence of degrading tail enzymes (24). Similarly, Romulus was able to degrade biofilms of PS47 by 34.4% at the end of 24 h at concentrations equal to 10^9^ PFU/mL (25), although the exact mechanisms associated with this degradation are unknown.

At the outset of this study, our fundamental question was whether certain combinations of phages and antibiotics could improve biofilm clearance, given the prevalence of biofilms in difficult-to-treat pathological states such as prosthetic bone and joint infections (26). Our intention to use a phage cocktail in these experiments was based on frequent reports proposing cocktails for broadening activity and reducing resistance in phage therapy. We therefore set out to identify a cocktail that showed evidence of these properties. This was done through a series of plaque assays, planktonic growth assays, and cross-resistance tests with spontaneously phage-resistant mutants. Assessing these experiments, the following themes emerged.

### Phage sensitivity in the planktonic state is not necessarily translatable to phage sensitivity in the biofilm state.

Plaque assay results did not always match phage activity in broth, but the results were often similar. In contrast, much bigger differences were observed when we transitioned from plaque assay and planktonic time-kill experiments to experiments in the biofilm state. For instance, DNS-VISA isolate D712 was highly susceptible to Intesti13 in plaque and planktonic assays but nearly unaffected in biofilm assays unless combined with daptomycin and ceftaroline. A similar pattern was observed for MRSA strain 8014. Phage Intesti13 was not effective against this strain in the biofilm state at concentrations as high as 10^7^. Differing phage sensitivities of planktonic and biofilm bacteria have been reported many times (21, 27), and our data affirm the importance of incorporating this consideration into the design and testing of antibacterial regimens.

### Phage concentration makes a difference both in single treatments and in combination with antibiotics.

Although phages are sometimes discussed as being self-dosing entities (28), the input concentration of phage in 24-h time-kill wells made a difference. For instance, phage Intesti13 did not clear the bacterial biofilms at starting concentration of 10^6^, while it caused clearance at 10^7^. This concentration-based killing pattern was also observed in combinations with antibiotics or the two other phages. Dose dependency was previously reported in rodents and chickens where minimum effective phage concentration was 10^7^ to 10^9^ PFU/animal (14, 29–31). Considering the fact that the biofilm matrix can contain phage-inactivating enzymes (32), perhaps low phage concentrations (<10^7^ in this case) are being inactivated before infecting the bacteria and replicating (32). Another challenge regarding the biofilm environment is that diffusion inside the biofilm matrix and phage entrapment may not lead to successful infection (23, 33, 34).

### Phage-antibiotic combinations can enhance efficacy.

Phage-antibiotic combinations led to eradication of biofilms at lower phage concentrations than by phage alone. Antimicrobial synergy of phage-antibiotic combinations was previously reported for various strains (9, 35). Comeau et al. used the term phage-antibiotic synergy, or PAS, for the first time in 2007 (36, 37). They reported larger phage plaques and increased phage production after exposure to sublethal amounts of antibiotics. Although we did not observe increased phage production in the presence of antibiotics (8), we noticed enhanced suppression of bacterial biofilms in phage-antibiotic regimens at lower phage concentrations.

### Antibiotics in combination with single phages versus cocktails.

Our results highlight the details regarding the breakdown of single phages versus double- and triple-phage cocktails. We selected a cocktail containing phages Intesti13, Romulus, and Sb-1 based on our initial host range screening experiments and the frequency of resistance obtained from our resistance tests. However, biofilm experiments with DNS-VISA strain D712 and MRSA strain 8014 showed that the Intesti13 phage in combination with antibiotics (Fig. 4) was as effective as the 3-phage cocktail in combination with antibiotics. The combination of any two phages did not demonstrate enhancement in comparison to Intesti13 alone or the three-phage cocktail (data not shown). For these two strains, the phage cocktail did not add value beyond the activity of the best single phage. However, we expect that the cocktail would be important for achieving antibiofilm activity across a wider range of strains. In addition, our planktonic growth curves showed that some strains of S. aureus can be more susceptible to the cocktail than to any of its component phages, which might also sometimes occur in biofilms. Finally, we hypothesize that phage resistance might arise less often with the cocktail than with single phages. Of note, we did not observe resistance to Romulus in the cocktail treatments, while resistance was very frequent with Romulus alone or Romulus-antibiotic regimens (data not shown).

This study has several limitations. It was based on static experiments and plaque assays without humanized dose testing or dynamic pharmacokinetic/pharmacodynamic models. It is important to investigate phage-antibiotic efficacy in a dynamic setting mimicking release rates similar to the human body (38). Also, biofilm age is reported as a key factor for phage diffusion and efficacy (33). Here, we only worked with biofilms formed over the course of 18 to 24 h, and we did not investigate biofilms with various maturity profiles. Additionally, our final biofilm experiments were performed only with two isolates. We did not investigate specific phage parameters such as burst size, attachment rate, and latent period in the presence of antibiotics. While based on previous literature, sublethal amounts of antibiotics can potentially enhance attachment rate and increase burst size (37). Our experiments only incorporate the aggregate effects of these factors. As we collect data from more strains and in more complex models, we hope to learn more about the importance of specific phage traits when determining the effects of adjunctive phage-antibiotic regimens.

## MATERIALS AND METHODS

### Antimicrobial agents and media.

Daptomycin was purchased from Merck & Co., Inc, (Whitehouse Station, NJ), and ceftaroline powder was provided by AbbVie, Inc. (North Chicago, IL). Colony counts were determined using tryptic soy agar (TSA). Mueller-Hinton broth II (MHB) (Difco, Detroit, MI, USA) with 25 mg/L calcium and 12.5 mg/L magnesium was used for susceptibility testing. For all experiments with daptomycin, an additional 25 mg/L of calcium was added to the broth due to the dependency of daptomycin on calcium for antimicrobial activity. Phage propagation and testing were done using heart infusion broth (HIB; BD, Bacto, San Jose, CA, USA) with 1.5% agar (Oxoid, Lenexa, KS, USA) for underlays and 0.7% agar for overlays. Tryptic soy broth (TSB; Difco, Detroit, MI) supplemented with 1% glucose (GSTSB) was used for biofilm assays. Salt magnesium buffer (SMB; Boston Bioproducts; catalog no. BM-342) was used for all the dilutions.

### Bacterial strains.

Seventy-two well-characterized S. aureus strains were used for initial phage host range screening. An additional 8 clinical MRSA strains were selected for additional cocktail selection experiments, biofilm formation quantification, and antibiofilm activity experiments. These consisted of three clinical strain pairs, with each pair, including an MRSA or hVISA parent and a mutant, having the DNS-VISA phenotype. These strain pairs used are patient derived, meaning that the parent isolate was recovered from a patient upon hospital admission and the mutant isolate was isolated after a period of antibiotic exposure; the mutants were subsequently confirmed to be descendants of the parent. The characteristics of these strains are shown in Fig. 1 and Table 1.

### Bacteriophages, source, and amplification.

All phages used in this study belong to the *Herelleviridae* family and *Twortvirinae* subfamily, as defined in ICTV Master Species List no. 37. Phage K was obtained from ATCC (39, 40) and grown on S. aureus strain ATCC 19685. Sb-1 (41) and Intesti13 are myophages belonging to the *Kayvirus* genus that were isolated from bacteriophage solutions purchased from Eliava Institute (Tbilisi, Georgia); they were grown on S. aureus D712 and ATCC 19685, respectively. Stab21 was isolated in Albania and also belongs to the *Kayvirus* genus (42); it was grown on Staphylococcus xylosus 6743. Romulus was isolated in Belgium and belongs to the *Silviavirus* genus (43); it was grown on S. aureus PS47. The genome sequences and biological characteristics of phages K, Stab21, and Romulus are described in detail in the cited references. All of these phages possess long, fixed terminal repeats in their packaged form and are therefore unlikely to mediate transduction. Our isolates of phages Sb-1 and Intesti13 were 96.7% and 96.8% identical to phage K, respectively (see Fig. S1 in the supplemental material). Our isolate of Sb-1 reflects the sequence reported by Sergueev et al. (GenBank accession no. MN336262) (44) rather than the sequence reported in 2010 (GenBank accession no. HQ163896), although our isolate contains more copies of the region 1 iteron repeat than the isolates described by Sergueev et al.

Phages were propagated to obtain high-titer stocks to use in resistance testing and time-kill analyses. To begin, an underlay layer of 1.5% HIB agar was poured into square petri plates. A 6-mL overlay of 0.7% HIB agar was immediately combined with 100 μL of an overnight host S. aureus bacterial culture containing approximately 10^9^ CFU/mL and poured atop the underlay layer. The overlay was briefly allowed to set, and following this, 750 μL of purified bacteriophage was spread over the top and incubated in a 37°C incubator overnight. The overlay agar was scraped into 3 mL of phosphate-buffered saline (PBS) plus 10 mM magnesium sulfate and centrifuged at 1,000 rpm for 25 min at 4°C. The supernatant was filtered and stored covered at 2 to 8°C for experimental use. All phage stocks have been sequenced to confirm their identities.

### Antibiotic susceptibility testing.

Minimum biofilm inhibitory concentration (MBIC) values were determined in duplicate using the pin-lid method (formerly referred to as the Calgary biofilm device) (45, 46). Briefly, biofilms were grown on plastic pins for 18 to 24 h followed by antibiotic susceptibility testing via the broth microdilution method (BMD) following Clinical and Laboratory Standards Institute (CLSI) guidelines (47). Combination MBIC values for DAP in the presence of CPT were determined by supplementing the broth with concentrations of CPT at 0.5× or 1× MBIC. Table S4 shows the MIC and MBIC values for the two isolates used in final time-kill experiments.

### Phage activity assays.

Phage activity was tested using the (i) plate-based method (efficiency of plating), and (ii) broth-based method. The plate-based method was performed as reported previously (11, 14, 38). Briefly, 100 μL of a 16- to 18-h HIB culture was transferred to a 14-mL snap-cap tube, 6 mL of molten HIB (melted and then held at 50°C) was added to the tube, and then the tube contents were poured evenly over a 100- by 100-mm square Hearth infusion broth agar (HIBA) plate. Tenfold serial dilutions of each phage were spotted onto the overlays. Plaques were counted after 20 to 24 h of incubation at 37°C.

The broth-based method compared 48-h bacterial growth curves in the presence or absence of phage using a BioTek LogPhase 600 plate reader with incubation at 37°C and orbital shaking at 500 rpm except during optical density at 600 nm (OD_600_) readings, which were collected every 20 min. Growth curves were constructed using 96-well, U-bottom, non-tissue-culture-treated plates containing ~10^6^ CFU/well at time zero. Wells with phage initially contained ~10^5^ PFU or 10^4^ PFU per well. HIB was used instead of phage in bacteria-only control wells. An automated blank adjustment was applied to all OD_600_ readings using the Gen5 software.

### BIM generation.

This experiment used ATCC 19685 and three DNS-VISA strains. In order to isolate bacteriophage-insensitive mutants (BIMs) that spontaneously arise in the absence of phages, a single colony of each strain was picked into 10 mL of HIB broth and incubated at 37°C on a shaker at 200 rpm. When the cultures reached an OD_600_ of approximately 0.8, 100 μL of the bacterial culture was mixed with 100 μL of concentrated phage and left to sit for 10 min at room temperature. Then, 3 mL molten HIBA was added to each sample, and the mixture was poured over a round HIBA plate. Plates were incubated for 48 h at 37°C. The number of colonies on each plate was recorded. Up to 8 BIMs per plate were picked and subcultured onto HIBA in order to separate the bacteria from residual phages on the overlay plate. BIMs that could be recovered in this way were retested for phage sensitivity using the spot dilution method.

### Biofilm formation assay.

Biofilm formation quantification was carried out in clear flat-bottom 96-well plates as reported previously (48). Briefly, in order to produce biofilms, stationary-phase cultures were inoculated to the fresh culture medium (1:100 ratio). All samples were assayed in triplicate to ensure reproducibility. We transferred 200 μL of this culture into a 96-well microtiter plate. Blank wells with fresh broth (not inoculated) were included as control. The 96-well plate was incubated in a shaker incubator for 16 to 18 h. After incubation and biofilm formation, planktonic bacteria were discarded, and the adherent bacteria (biofilms) were stained with 125 μL of 0.1% crystal violet solution. The plate was inverted to the waste tray to remove liquid and then washed with water. The best washing results were achieved by submerging the plate in the water so the biofilm structure remained intact. To allow evaporation of excess water, the 96-well plate was left to dry for 6 h. One hundred fifty microliters of 30% acetic acid was added to each well to solubilize the biofilm and mixed by pipetting in and out. The optical density of all samples was measured at 550 nm.

### Antibiofilm activity experiments.

Antibiofilm activity was tested using daptomycin, vancomycin, and ceftaroline at 0.5× MBIC or 1× MBIC values to simulate conditions under which antibiotic treatment of these strains would fail. Rifampin was used at peak concentration since the MBICs to rifampin were higher than peak (free maximum concentration of drug in serum [*fC*_max_] = 0.002) for all strains. The concentration of phage in time-kill plates ranged between 10 and 10^7^ PFU/well. The experiment was performed in duplicate in 24-well tissue culture-treated plates with 2 mL of broth and 4 sterile polystyrene beads (3 mm; Fisher Scientific) in each well. Plates were incubated in a shaker incubator at 37°C for 24 h to allow for biofilm growth on the beads in 1% glucose-supplemented tryptic soy broth (GSTSB). After 24 h of incubation, GSTSB was aspirated and replaced with MHB. Antibiotic and phage were then added. Plates were incubated for 18 to 24 h of growth at 37°C, and, depending on the turbidity of the plate (based on initial screening, we used an optical density of 0.4 as our cutoff value after normalization with media control wells), the corresponding treatment was characterized as successful inhibition of biofilms versus no effect.

## References

[1] Klevens RM, Edwards JR, Richards CL, Horan TC, Gaynes RP, Pollock DA, Cardo DM. 2007. Estimating health care-associated infections and deaths in U.S. hospitals, 2002. Public Health Rep 122:160–166. doi:10.1177/003335490712200205. 17357358 PMC1820440

[2] Guidos RJ. 2011. Combating antimicrobial resistance: policy recommendations to save lives. Clin Infect Dis 52:S397–S428. doi:10.1093/cid/cir153. 21474585 PMC3738230

[3] Thompson T. 2022. The staggering death toll of drug-resistant bacteria. Nature doi:10.1038/d41586-022-00228-x. 35102288

[4] Suh GA, Lodise TP, Tamma PD, Knisely JM, Alexander J, Aslam S, Barton KD, Bizzell E, Totten KMC, Campbell JL, Chan BK, Cunningham SA, Goodman KE, Greenwood-Quaintance KE, Harris AD, Hesse S, Maresso A, Nussenblatt V, Pride D, Rybak MJ, Sund Z, van Duin D, Van Tyne D, Patel R, for the Antibacterial Resistance Leadership Group. 2022. Considerations for the use of phage therapy in clinical practice. Antimicrob Agents Chemother 66:e02071-21. doi:10.1128/aac.02071-21. 35041506 PMC8923208

[5] Labrie SJ, Samson JE, Moineau S. 2010. Bacteriophage resistance mechanisms. Nat Rev Microbiol 8:317–327. doi:10.1038/nrmicro2315. 20348932

[6] Chan BK, Abedon ST, Loc-Carrillo C. 2013. Phage cocktails and the future of phage therapy. Future Microbiol 8:769–783. doi:10.2217/fmb.13.47. 23701332

[7] Oechslin F. 2018. Resistance development to bacteriophages occurring during bacteriophage therapy. Viruses 10:351. doi:10.3390/v10070351. 29966329 PMC6070868

[8] Kebriaei R, Lev KL, Shah RM, Stamper KC, Holger DJ, Morrisette T, Kunz Coyne AJ, Lehman SM, Rybak MJ. 2022. Eradication of biofilm-mediated methicillin-resistant Staphylococcus aureus infections in vitro: bacteriophage-antibiotic combination. Microbiol Spectr 10:e00411-22. doi:10.1128/spectrum.00411-22. 35348366 PMC9045164

[9] Li X, He Y, Wang Z, Wei J, Hu T, Si J, Tao G, Zhang L, Xie L, Abdalla AE, Wang G, Li Y, Teng T. 2021. A combination therapy of phages and antibiotics: two is better than one. Int J Biol Sci 17:3573–3582. doi:10.7150/ijbs.60551. 34512166 PMC8416725

[10] Diallo K, Dublanchet A. 2022. Benefits of combined phage–antibiotic therapy for the control of antibiotic-resistant bacteria: a literature review. Antibiotics (Basel) 11:839. doi:10.3390/antibiotics11070839. 35884092 PMC9311689

[11] Kebriaei R, Lev K, Morrisette T, Stamper KC, Abdul-Mutakabbir JC, Lehman SM, Morales S, Rybak MJ. 2020. Bacteriophage-antibiotic combination strategy: an alternative against methicillin-resistant phenotypes of Staphylococcus aureus. Antimicrob Agents Chemother 64:e00461-20. doi:10.1128/AAC.00461-20. 32393490 PMC7318012

[12] Van Nieuwenhuyse B, Van der Linden D, Chatzis O, Lood C, Wagemans J, Lavigne R, Schroven K, Paeshuyse J, de Magnée C, Sokal E, Stéphenne X, Scheers I, Rodriguez-Villalobos H, Djebara S, Merabishvili M, Soentjens P, Pirnay J-P. 2022. Bacteriophage-antibiotic combination therapy against extensively drug-resistant Pseudomonas aeruginosa infection to allow liver transplantation in a toddler. Nat Commun 13:5725. doi:10.1038/s41467-022-33294-w. 36175406 PMC9523064

[13] Altamirano FLG, Kostoulias X, Subedi D, Korneev D, Peleg AY, Barr JJ. 2022. Phage-antibiotic combination is a superior treatment against Acinetobacter baumannii in a preclinical study. eBioMedicine 80:104045. doi:10.1016/j.ebiom.2022.104045. 35537278 PMC9097682

[14] Lehman SM, Mearns G, Rankin D, Cole RA, Smrekar F, Branston SD, Morales S. 2019. Design and preclinical development of a phage product for the treatment of antibiotic-resistant Staphylococcus aureus infections. Viruses 11:88. doi:10.3390/v11010088. 30669652 PMC6356596

[15] Tzipilevich E, Pollak-Fiyaksel O, Shraiteh B, Ben-Yehuda S. 2022. Bacteria elicit a phage tolerance response subsequent to infection of their neighbors. EMBO J 41:e109247. doi:10.15252/embj.2021109247. 34878184 PMC8804946

[16] Alves DR, Gaudion A, Bean JE, Perez Esteban P, Arnot TC, Harper DR, Kot W, Hansen LH, Enright MC, Jenkins ATA. 2014. Combined use of bacteriophage K and a novel bacteriophage to reduce Staphylococcus aureus biofilm formation. Appl Environ Microbiol 80:6694–6703. doi:10.1128/AEM.01789-14. 25149517 PMC4249044

[17] Ajuebor J, Buttimer C, Arroyo-Moreno S, Chanishvili N, Gabriel EM, O’Mahony J, McAuliffe O, Neve H, Franz C, Coffey A. 2018. Comparison of Staphylococcus phage K with close phage relatives commonly employed in phage therapeutics. Antibiotics 7:37. doi:10.3390/antibiotics7020037. 29693603 PMC6022877

[18] Lehman SM, Kongari R, Glass AM, Koert M, Ray MD, Plaut RD, Stibitz S. 2022. Phage K gp102 drives temperature-sensitive antibacterial activity on USA300 MRSA. Viruses 15:17. doi:10.3390/v15010017. 36680060 PMC9861931

[19] Moller AG, Lindsay JA, Read TD. 2019. Determinants of phage host range in Staphylococcus species. Appl Environ Microbiol 85:e00209-19. doi:10.1128/AEM.00209-19. 30902858 PMC6532042

[20] Kelly D, McAuliffe O, Ross RP, Coffey A. 2012. Prevention of Staphylococcus aureus biofilm formation and reduction in established biofilm density using a combination of phage K and modified derivatives. Lett Appl Microbiol 54:286–291. doi:10.1111/j.1472-765X.2012.03205.x. 22251270

[21] Abedon ST. 2015. Ecology of anti-biofilm agents II: bacteriophage exploitation and biocontrol of biofilm bacteria. Pharmaceuticals (Basel) 8:559–589. doi:10.3390/ph8030559. 26371011 PMC4588183

[22] Abedon ST. 2012. Spatial vulnerability: bacterial arrangements, microcolonies, and biofilms as responses to low rather than high phage densities. Viruses 4:663–687. doi:10.3390/v4050663. 22754643 PMC3386622

[23] Azeredo J, Sutherland IW. 2008. The use of phages for the removal of infectious biofilms. Curr Pharm Biotechnol 9:261–266. doi:10.2174/138920108785161604. 18691087

[24] Tkhilaishvili T, Lombardi L, Klatt A-B, Trampuz A, Di Luca M. 2018. Bacteriophage Sb-1 enhances antibiotic activity against biofilm, degrades exopolysaccharide matrix and targets persisters of Staphylococcus aureus. Int J Antimicrob Agents 52:842–853. doi:10.1016/j.ijantimicag.2018.09.006. 30236955

[25] Gutiérrez D, Rodríguez-Rubio L, Martínez B, Rodríguez A, García P. 2016. Bacteriophages as weapons against bacterial biofilms in the food industry. Front Microbiol 7:825. doi:10.3389/fmicb.2016.00825. 27375566 PMC4897796

[26] Hofstee MI, Muthukrishnan G, Atkins GJ, Riool M, Thompson K, Morgenstern M, Stoddart MJ, Richards RG, Zaat SAJ, Moriarty TF. 2020. Current concepts of osteomyelitis: from pathologic mechanisms to advanced research methods. Am J Pathol 190:1151–1163. doi:10.1016/j.ajpath.2020.02.007. 32194053

[27] Abedon ST. 2017. Phage “delay” towards enhancing bacterial escape from biofilms: a more comprehensive way of viewing resistance to bacteriophages. AIMS Microbiol 3:186–226. doi:10.3934/microbiol.2017.2.186. 31294157 PMC6605007

[28] Loc-Carrillo C, Abedon ST. 2011. Pros and cons of phage therapy. Bacteriophage 1:111–114. doi:10.4161/bact.1.2.14590. 22334867 PMC3278648

[29] Majewska J, Beta W, Lecion D, Hodyra-Stefaniak K, Kłopot A, Kaźmierczak Z, Miernikiewicz P, Piotrowicz A, Ciekot J, Owczarek B, Kopciuch A, Wojtyna K, Harhala M, Mąkosa M, Dąbrowska K. 2015. Oral application of T4 phage induces weak antibody production in the gut and in the blood. Viruses 7:4783–4799. doi:10.3390/v7082845. 26308042 PMC4576206

[30] Wolochow H, Hildebrand GJ, Lamanna C. 1966. Translocation of microorganisms across the intestinal wall of the rat: effect of microbial size and concentration. J Infect Dis 116:523–528. doi:10.1093/infdis/116.4.523. 4959185

[31] Abedon ST, García P, Mullany P, Aminov R. 2017. Editorial: phage therapy: past, present and future. Front Microbiol 8:981. doi:10.3389/fmicb.2017.00981. 28663740 PMC5471325

[32] Ferriol-González C, Domingo-Calap P. 2020. Phages for biofilm removal. Antibiotics (Basel) 9:268. doi:10.3390/antibiotics9050268. 32455536 PMC7277876

[33] Abedon ST. 2016. Bacteriophage exploitation of bacterial biofilms: phage preference for less mature targets? FEMS Microbiol Lett 363:fnv246. doi:10.1093/femsle/fnv246. 26738755

[34] Romaní AM, Fund K, Artigas J, Schwartz T, Sabater S, Obst U. 2008. Relevance of polymeric matrix enzymes during biofilm formation. Microb Ecol 56:427–436. doi:10.1007/s00248-007-9361-8. 18227962

[35] Ryan EM, Alkawareek MY, Donnelly RF, Gilmore BF. 2012. Synergistic phage-antibiotic combinations for the control of Escherichia coli biofilms in vitro. FEMS Immunol Med Microbiol 65:395–398. doi:10.1111/j.1574-695X.2012.00977.x. 22524448

[36] Comeau AM, Tétart F, Trojet SN, Prère M-F, Krisch HM. 2007. Phage-antibiotic synergy (PAS): beta-lactam and quinolone antibiotics stimulate virulent phage growth. PLoS One 2:e799. doi:10.1371/journal.pone.0000799. 17726529 PMC1949050

[37] Kim M, Jo Y, Hwang YJ, Hong HW, Hong SS, Park K, Myung H. 2018. Phage-antibiotic synergy via delayed lysis. Appl Environ Microbiol 84:e02085-18. doi:10.1128/AEM.02085-18. 30217844 PMC6210123

[38] Kebriaei R, Lev KL, Stamper KC, Lehman SM, Morales S, Rybak MJ. 2020. Bacteriophage AB-SA01 cocktail in combination with antibiotics against MRSA-VISA strain in an in vitro pharmacokinetic/pharmacodynamic model. Antimicrob Agents Chemother 65:e01863-20. doi:10.1128/AAC.01863-20. 33077648 PMC7927802

[39] O'Flaherty S, Coffey A, Edwards R, Meaney W, Fitzgerald GF, Ross RP. 2004. Genome of staphylococcal phage K: a new lineage of Myoviridae infecting Gram-positive bacteria with a low G+C content. J Bacteriol 186:2862–2871. doi:10.1128/JB.186.9.2862-2871.2004. 15090528 PMC387793

[40] Gill JJ. 2014. Revised genome sequence of Staphylococcus aureus bacteriophage K. Genome Announc 2:e01173-13. doi:10.1128/genomeA.01173-13. PMC3900892 24459260

[41] Kvachadze L, Balarjishvili N, Meskhi T, Tevdoradze E, Skhirtladze N, Pataridze T, Adamia R, Topuria T, Kutter E, Rohde C, Kutateladze M. 2011. Evaluation of lytic activity of staphylococcal bacteriophage Sb-1 against freshly isolated clinical pathogens. Microb Biotechnol 4:643–650. doi:10.1111/j.1751-7915.2011.00259.x. 21481199 PMC3819013

[42] Oduor JMO, Kadija E, Nyachieo A, Mureithi MW, Skurnik M. 2020. Bioprospecting Staphylococcus phages with therapeutic and bio-control potential. Viruses 12:133. doi:10.3390/v12020133. 31979276 PMC7077315

[43] Vandersteegen K, Kropinski AM, Nash JHE, Noben J-P, Hermans K, Lavigne R. 2013. Romulus and Remus, two phage isolates representing a distinct clade within the Twortlikevirus genus, display suitable properties for phage therapy applications. J Virol 87:3237–3247. doi:10.1128/JVI.02763-12. 23302893 PMC3592175

[44] Sergueev KV, Filippov AA, Farlow J, Su W, Kvachadze L, Balarjishvili N, Kutateladze M, Nikolich MP. 2019. Correlation of host range expansion of therapeutic bacteriophage Sb-1 with allele state at a hypervariable repeat locus. Appl Environ Microbiol 85:e01209-19. doi:10.1128/AEM.01209-19. 31492663 PMC6821964

[45] Ceri H, Olson ME, Stremick C, Read RR, Morck D, Buret A. 1999. The Calgary biofilm device: new technology for rapid determination of antibiotic susceptibilities of bacterial biofilms. J Clin Microbiol 37:1771–1776. doi:10.1128/JCM.37.6.1771-1776.1999. 10325322 PMC84946

[46] Macià MD, Rojo-Molinero E, Oliver A. 2014. Antimicrobial susceptibility testing in biofilm-growing bacteria. Clin Microbiol Infect 20:981–990. doi:10.1111/1469-0691.12651. 24766583

[47] Clinical and Laboratory Standards Institute. 2020. Performance standards for antimicrobial susceptibility testing, 30th ed. M100. Clinical and Laboratory Standards Institute, Wayne, PA.

[48] Coffey BM, Anderson GG. 2014. Biofilm formation in the 96-well microtiter plate, p 631–641. *In* Filloux A, Ramos J-L (ed), Pseudomonas methods and protocols. Springer, New York, NY. 10.1007/978-1-4939-0473-0_48 24818938

