# Supplementary material for: Optimization of Phage-Antibiotic Combinations against Staphylococcus aureus Biofilms
Source: Microbiol Spectr. 2023 May 18;11(3):e04918-22. doi: 10.1128/spectrum.04918-22 (PMC10269792; doi:10.1128/spectrum.04918-22)
Supplement: Supplemental file 1 — Supplemental material. Download spectrum.04918-22-s0001.pdf, PDF file, 0.4 MB [file spectrum.04918-22-s0001.pdf]

Supplementary Material

	Romulus (NC_020877)	Stab21 (LR215719)	Sb-1	Intesti13	K (NC_005880)
Romulus (NC_020877)	100	42.5	43.4	43.3	43.1
Stab21 (LR215719)		100	91.1	91.1	92.4
Sb-1			100	99.7	96.7
Intesti13				100	96.8
K (NC_005880)					100

Figure S1. Percent genome similarity of the five phages (constructed in VIRIDIC using single-copy genomes)

Supplementary Tables 1 through 3 show selected results of dilution spot assays conducted on the BIMs that were isolated in this study. Up to 8 BIMs per phage+host pairing was subcultured from their original isolation plate, re-grown in broth, and challenged in spot dilution assays. The red text "DNG on HIBA" means that no colonies could be recovered when this BIM was picked from the isolation plate and streaked on a fresh HIBA plate. Similarly, "DNG in HIB" indicates that this BIM could not be subsequently grown on broth, despite several attempts. These BIMs were therefore classified as being sensitive to the original challenge phage and are marked as S*. See the Figure 1 caption for explanation of color-coded cells.

Table S1. Phage Sensitivity of BIMs that were isolated against phage K

Isolated BIMs against Phage	*S. aureus* Strain	Phage Titer, log10(1+PFU/mL)				
		K	Sb-1	Intesti13	Stab21	Romulus
K	19685 (parental)	8.05	7.68	7.75	7.85	5.68
	BIM#1 (DNG on HIBA)	S*	-	-	-	-
K	D712 (parental)	7.86	7.41	8.12	7.38	0.00
	BIM#5 (DNG on HIBA)	S*	-	-	-	-
	BIM#3 (DNG on HIBA)	S*	-	-	-	-
	BIM#2	0.00	7.92	7.95	7.00	0.00
	BIM#4	0.00	6.60	6.78	0.00	0.00
	BIM#1	0.00	4.56	4.82	0.00	0.00
K	8015 (parental)	7.95	7.16	7.99	7.58	0.00
	BIM#1	7.79	7.97	7.79	7.38	0.00
	BIM#2	0.00	7.66	7.79	7.45	0.00
K	306 (parental)	8.11	7.38	7.92	7.89	7.58
	BIM#2	7.30	7.75	7.51	7.41	7.91
	BIM#1	2.60	2.90	0.00	0.00	6.99

Table S2. Phage sensitivity of BIMS that were isolated against phage Intesti13

Isolated BIMs against Phage	*S. aureus* Strain	Phage Titer, log10(1+PFU/mL)				
		K	Sb-1	Intesti13	Stab21	Romulus
Intesti13	19685 (parental)	8.05	7.68	7.75	7.85	5.68
	BIM#1	0.00	0.00	0.00	0.00	5.73
Intesti13	D712 (parental)	7.86	7.41	8.12	7.38	0.00
	BIM#4 (DNG in HIB)	NA	NA	S*	NA	NA
Intesti13	8015 (parental)	7.95	7.16	7.99	7.58	0.00
	BIM#1	7.58	7.79	7.70	7.53	0.00
Intesti13	306 (parental)	8.11	7.38	7.92	7.89	7.58
	BIM#8	7.90	7.30	7.30	7.38	7.90
	BIM#2	7.00	4.41	3.34	7.30	7.79
	BIM#3	6.60	4.48	3.15	7.08	7.81
	BIM#6	7.90	4.73	3.51	6.60	7.99
	BIM#7	7.30	4.90	3.78	7.30	7.86
	BIM#4	3.15	4.34	0.00	0.00	7.87
	BIM#5	2.90	3.90	3.00	2.60	8.01
	BIM#1	3.00	4.30	2.90	2.78	8.00

Table S3. Phage sensitivity of BIMs that were isolated against Romulus

Isolated BIMs against Phage	*S. aureus* Strain	Phage Titer, log10(1+PFU/mL)				
		K	Sb-1	Intesti13	Stab21	Romulus
Romulus	19685 (parental)	8.05	7.68	7.75	7.85	5.68
	BIM#5 (DNG on HIBA)	-	-	-	-	S*
	BIM#2	7.88	8.26	8.19	7.96	0.00
	BIM#3	8.00	8.16	8.27	7.76	0.00
	BIM#4	7.89	8.11	8.15	7.60	0.00
	BIM#6	8.11	8.41	8.41	7.76	0.00
	BIM#7	8.05	8.11	8.22	7.70	0.00
	BIM#8	8.08	8.66	8.45	3.51	3.51
	BIM#1	7.94	8.51	8.64	0.00	0.00

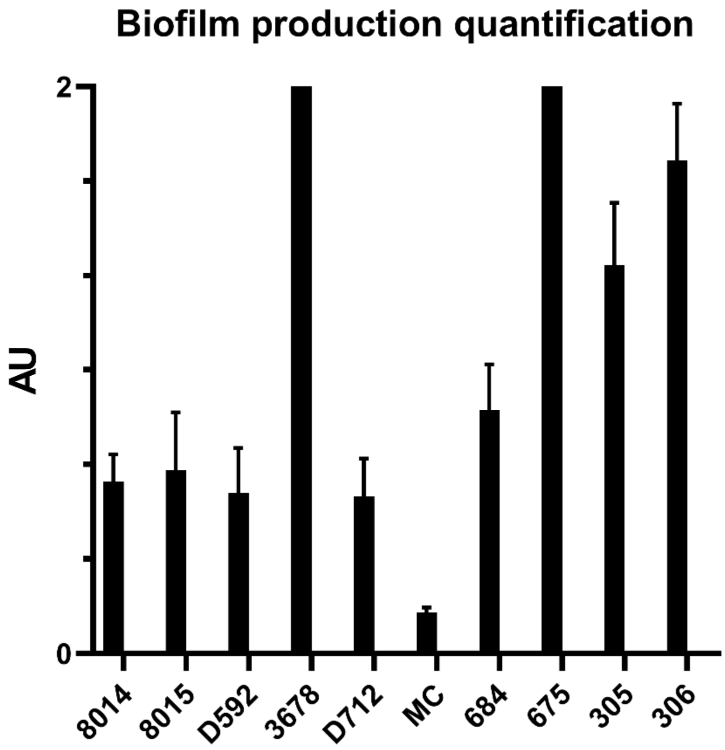

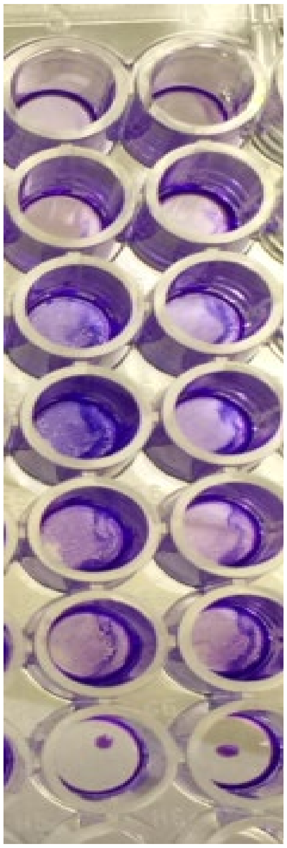

Figure S2. Biofilm formation by three strain pairs, relative to the *S. aureus* 3678 (reference biofilm forming strain ATCC 35556) positive control and media only (MC) negative control.

Table.S4: List of MIC values in planktonic and biofilm state.

Strain	D712		8014	
**Antibiotic**	**MIC (mg/L)**	**MBIC (mg/L)**	**MIC (mg/L)**	**MBIC (mg/L)**
**DAP**	4	8	0.5	8
**VAN**	4	8	2	8
**CPT**	0.5	4	1	1

.

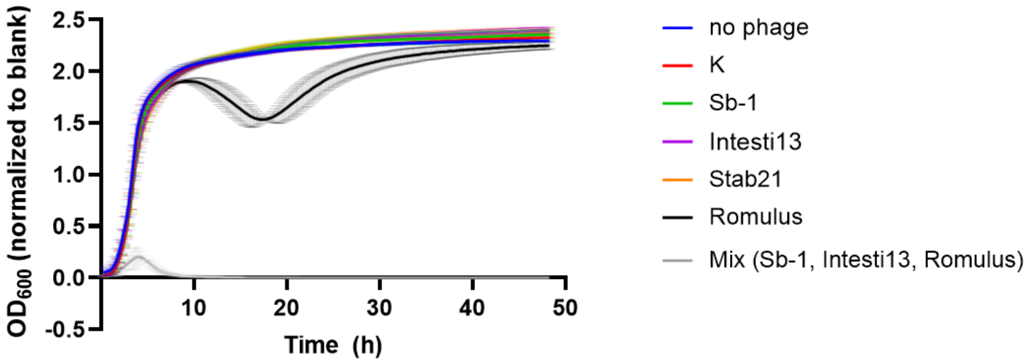

Figure S3. Planktonic growth of a Stab21-insensitive BIM of *S. aureus* 306 in the presence of single phages or a three-phage cocktail. Initial quantities of bacteria and phages were approximately 3×10^6 CFU and 1×10^5 PFU, respectively.
